# Improving internal model strength and performance of prosthetic hands using augmented feedback

**DOI:** 10.1186/s12984-018-0417-4

**Published:** 2018-07-31

**Authors:** Ahmed W. Shehata, Leonard F. Engels, Marco Controzzi, Christian Cipriani, Erik J. Scheme, Jonathon W. Sensinger

**Affiliations:** 1 Institute of Biomedical Engineering, University of New Brunswick, Fredericton, NB E3B 5A3 Canada; 2 Department of Electrical and Computer Engineering, University of New Brunswick, Fredericton, NB E3B 5A3 Canada; 3 Division of Physical Medicine and Rehabilitation, Department of Medicine, University of Alberta, Edmonton, AB T6G 2E1 Canada; 4 Scuola Superiore Sant’Anna, The BioRobotics Institute, V.le R. Piaggio 34, 56025 Pontedera, PI Italy

**Keywords:** Prosthetics, Electromyography, Support vector machines, Internal model, Motor learning, Performance, Muscles, Real-time systems, Augmented feedback, Sensory feedback

## Abstract

**Background:**

The loss of an arm presents a substantial challenge for upper limb amputees when performing activities of daily living. Myoelectric prosthetic devices partially replace lost hand functions; however, lack of sensory feedback and strong understanding of the myoelectric control system prevent prosthesis users from interacting with their environment effectively. Although most research in augmented sensory feedback has focused on real-time regulation, sensory feedback is also essential for enabling the development and correction of internal models, which in turn are used for planning movements and reacting to control variability faster than otherwise possible in the presence of sensory delays.

**Methods:**

Our recent work has demonstrated that audio-augmented feedback can improve both performance and internal model strength for an abstract target acquisition task. Here we use this concept in controlling a robotic hand, which has inherent dynamics and variability, and apply it to a more functional grasp-and-lift task. We assessed internal model strength using psychophysical tests and used an instrumented Virtual Egg to assess performance.

**Results:**

Results obtained from 14 able-bodied subjects show that a classifier-based controller augmented with audio feedback enabled stronger internal model (*p* = 0.018) and better performance (*p* = 0.028) than a controller without this feedback.

**Conclusions:**

We extended our previous work and accomplished the first steps on a path towards bridging the gap between research and clinical usability of a hand prosthesis. The main goal was to assess whether the ability to decouple internal model strength and motion variability using the continuous audio-augmented feedback extended to real-world use, where the inherent mechanical variability and dynamics in the mechanisms may contribute to a more complicated interplay between internal model formation and motion variability. We concluded that benefits of using audio-augmented feedback for improving internal model strength of myoelectric controllers extend beyond a virtual target acquisition task to include control of a prosthetic hand.

## Background

The seemingly simple and seamless way adult humans use their hands to grasp and manipulate objects is in fact the result of years of training during childhood, and of a sophisticated blend of feedforward and feedback control mechanisms [1]. The function of such an elegant system may be corrupted when neurological injuries interrupt the connections between the central nervous system (CNS) and the periphery, as in the case of upper limb amputation. In this case, myoelectric prostheses provide a solution to restore hand function by partially restoring the feedforward control mechanism [2]. This mechanism is influenced by two key factors. The first factor is the way the user intentions are decoded, which affects the robustness of control signals driving the prosthesis’ motors. The second factor is the human understanding of the system, which is modeled by the CNS and is known as the internal model [3]. The ability to accurately estimate the current state of the musculoskeletal system and properly integrate information from various sensory feedback forms to predict the future state is determined by the strength of the internal model developed [4]. For prosthesis users, this model is mismatched since their prosthetic device properties and control are very different from that of a normal limb, and therefore the need to develop a new internal model or adjust the current one is presumed.

For a representative motor task, such as grasp-and-lift, the brain refines and updates the internal model using multi-modal sensory feedback (tactile, visual, and auditory) during and after the movement [5]. Unlike unimpaired individuals, myoelectric prosthesis users have to rely more on visual feedback, which has been found to negatively affect performance, as users spend more time monitoring their prosthesis or the objects being manipulated [6]. This increased dependency on visual feedback is due to the lack of adequate sensory feedback from the prosthetic devices [7]. This deficiency contributes to an inability of users to fully adjust their internal model and is known to affect overall performance [8].

To address this deficiency, researchers have investigated ways of providing augmented sensory information using invasive and non-invasive methods [9, 10]. Several of the invasive methods show promise, including Targeted Sensory Reinnervation and stimulation of sensory peripheral nerves [11–15]. However, many prosthesis users prefer non-invasive methods that do not require surgical intervention [16, 17].

Researchers have correspondingly evaluated non-invasive sensory substitution methods to provide sensory information either through an alternate sensory channel or through the natural channel but in a different modality [9]. Vibro-tactile [18, 19], mechano-tactile [20], electrotactile [21–23], skin stretch [24], and auditory cues [25] are just some of the techniques that have been developed and assessed to provide prosthesis users with supplementary feedback. Although some studies have shown that augmented sensory feedback had little to no effect on performance [26], others have demonstrated the efficacy of augmented sensory feedback in enhancing motor control even for the same experimental procedure [9]. This conflict may arise in part because it is unclear how this augmented feedback affects internal model development and, ultimately, the performance.

One hypothesis is that feedback improves performance through the integration of feedback in a real-time manner during a movement, known as real-time regulation [27–29]. Many studies showed promising improvement in performance [30, 31], sense of embodiment [32], and prosthesis incorporation [33] when using feedback for real-time regulation; however the efficacy of the feedback methods used, such as resolution and latency, introduces a new challenge [34]. To overcome this challenge, Dosen et al. [35] proposed providing electromyography (EMG) biofeedback to the user through visual feedback. Their results showed that users were able to exploit the augmented visual biofeedback to improve their performance in a grasping task. In a follow-up study, Schweisfurth and colleagues [36] implemented the EMG biofeedback using a multichannel electrotactile interface to transmit discrete levels of myoelectric signals to users. They compared this feedback approach to classic force feedback and found that the electrotactile biofeedback allowed for more predictable control and improved performance. However, it is unclear whether this improvement is driven by the use of this feedback for real-time regulation or by the adjustments made to the internal model.

Our group has recently suggested a framework that demonstrates that the strength of the internal model is indeed affected by feedback [37]. In the field of myoelectric prosthesis control, we used this framework to assess the strength of the internal model developed for able-bodied subjects when using different myoelectric control strategies. A series of tests were conducted to extract parameters that are used in this framework to compute uncertainties in the developed internal model. One test quantified the ability of subjects to use feedback to adapt and modify their control signals. Other tests quantified variability in control signals for a given controller and variability in the provided feedback. These parameters were used in this framework to determine a weighted factor of the feedback that is assumed to be combined with the internal model based on the uncertainty of the feedback.

In a previous study [38], we noted that various types of control strategies, in the very act of filtering biological signals (i.e., movement classification and activation thresholds), provide inherently different levels of visual biofeedback to the user. For instance, classification-based control provides no visual feedback about any class except the one it deems to be the correct class, thus denying the user of any knowledge about partial activations of other classes [39]. Whereas most research has focused on the impact of those filters on the control (motor) performance of the prosthesis (see reviews [40, 41]), we demonstrated that it also affects the ability of the person to form an internal model. In that study, we assessed the internal model strength and performance when using two common myoelectric control strategies [39, 42] that differed in the inherent feedback provided to the user, namely: (a) regression-based control or (b) classification-based control.

For a two DOF task, a regression-based control provides users with proportional feedback about activations of both DOF while a classification-based control provides users with feedback about only one dominant DOF at a time. We showed that the inclusion of information about the smaller modulations in the secondary DOF in regression controllers (unfiltered control signals) provided valuable and rich information to improve the internal model, even though it resulted in worse short-term performance as measured using task accuracy and path efficiency. In contrast, the inherent filter in classification-based control, which limited the control signal variability and thus improved the smoothness of movements, also prevented the formation of a strong internal model. In other words, continuous feedback-rich control strategies may be used to improve internal model strength, but classification-based controllers enable better immediate performance. Intrigued by this outcome and attempting to incorporate the benefits of both control strategies, in our next study we combined a classification-based control with a regression-based audio-augmented sensory feedback in a virtual target acquisition task [43]. Our outcomes demonstrated that this combination enabled both the development of a stronger internal model than the regression-based controller and better performance than the classification-based controller.

In the present study, we extended our previous work by investigating the benefits of using audio-augmented feedback when controlling a prosthetic hand. The main goal was to assess whether the ability to decouple internal model strength and motion variability, using the continuous audio-augmented feedback, extended to real-world use, where the inherent mechanical variability and dynamics in the mechanisms as well as the user-socket interfaces may contribute to a more complicated interplay between internal model formation and motion variability. To accomplish this goal, we compared internal model strength and performance of a classifier-based myoelectric controller with and without audio-augmented feedback during a grasp-and-lift task using a multi-degree of freedom (DOF) research prosthetic hand [44]. We assessed the internal model strength using psychophysical tests and used an instrumented Virtual Egg to assess the performance [38, 45]. Our results from 14 able-bodied subjects show that audio-augmented feedback may indeed be used to improve internal model strength and performance of a myoelectric prosthesis. These improvements may increase reliability and promote acceptance of prosthetic devices by powered prosthesis users.

## Methods

**Fig. 1:**
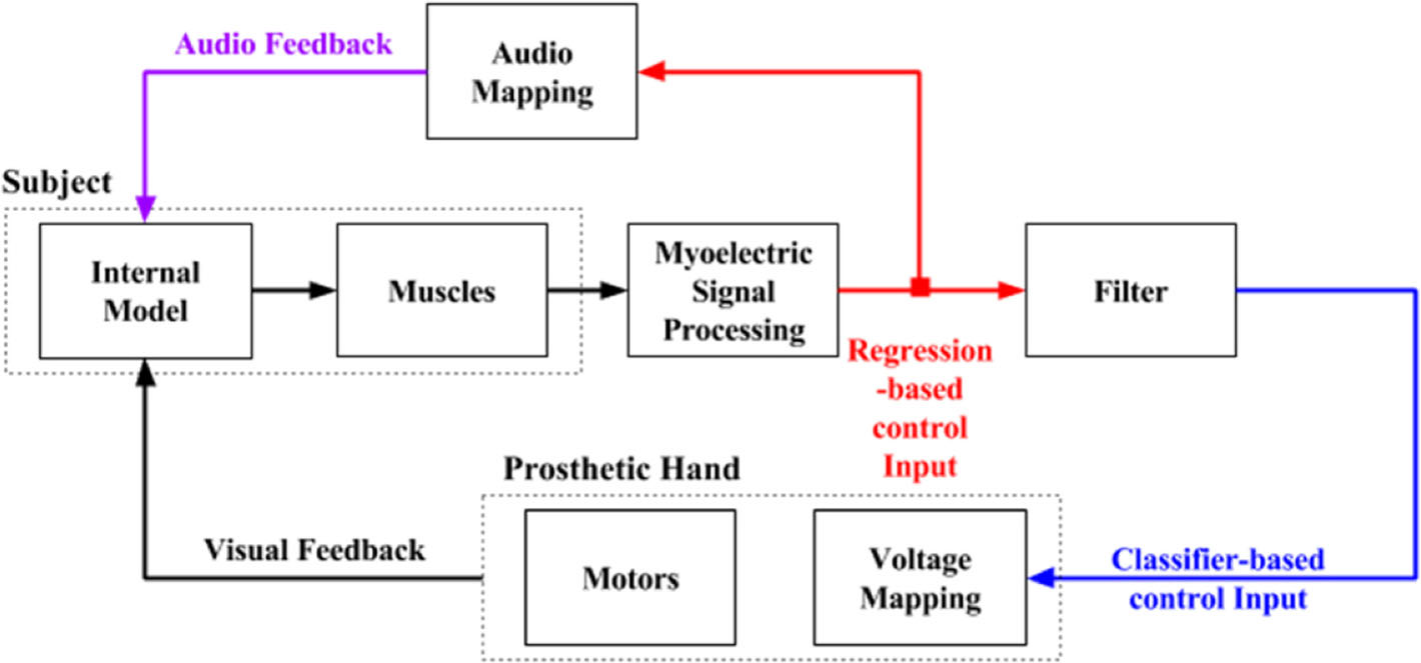
Closing the control loop using audio to augment the visual feedback. Dark blue lines represent the classifier-based control signals, red lines represent the regression-based control signals, and purple lines represent the audio feedback

Classifier-based myoelectric control is considered as one of the more advanced strategies of myocontrol [42] and may be implemented using various pattern recognition algorithms [46, 47]. In recent studies, we used a Support Vector Regression (SVR) algorithm, which has been proven to enable better performance than other algorithms, to implement a classifier-based myoelectric control strategy [38, 43]. This algorithm provided regression-based control signals that simultaneously activated more than 1 DOF at a time, which were subsequently gated to only allow the activation of 1 DOF at a time. In this work, we used these same gated, i.e., classifier-based control, signals to activate either hand open/close or thumb adduction/abduction of a prosthetic hand. Building on the classifier-based control, we implemented a novel control strategy, namely Audio-augmented Feedback control, which is able to effectively decouple internal model formation from control variability. We relayed information in the regression-based control signals through continuous auditory cues to augment the feedback from the classifier-based myoelectric control (Fig. 1). The amplitude of the audio feedback was directly proportional to the amplitude of the control signals. For each DOF, two distinct frequencies were assigned: open/close hand had 500/400 Hz assigned and thumb adduction/abduction had 900/800 Hz assigned.

### Participants

14 healthy subjects (8 male, and 6 female; mean and standard deviation of age: 25 ± 4.5 years) participated in this study. All participants had either normal or corrected-to-normal vision, were right-handed, and none had earlier experience with myoelectric pattern recognition control. Written informed consent according to the University of New Brunswick Research and Ethics Board and to Scuola Superiore Sant’Anna Ethical Committee was obtained from subjects before conducting the experiment (UNB REB 2014–019 and SSSA 02/2017).

### Setup

**Fig. 2:**
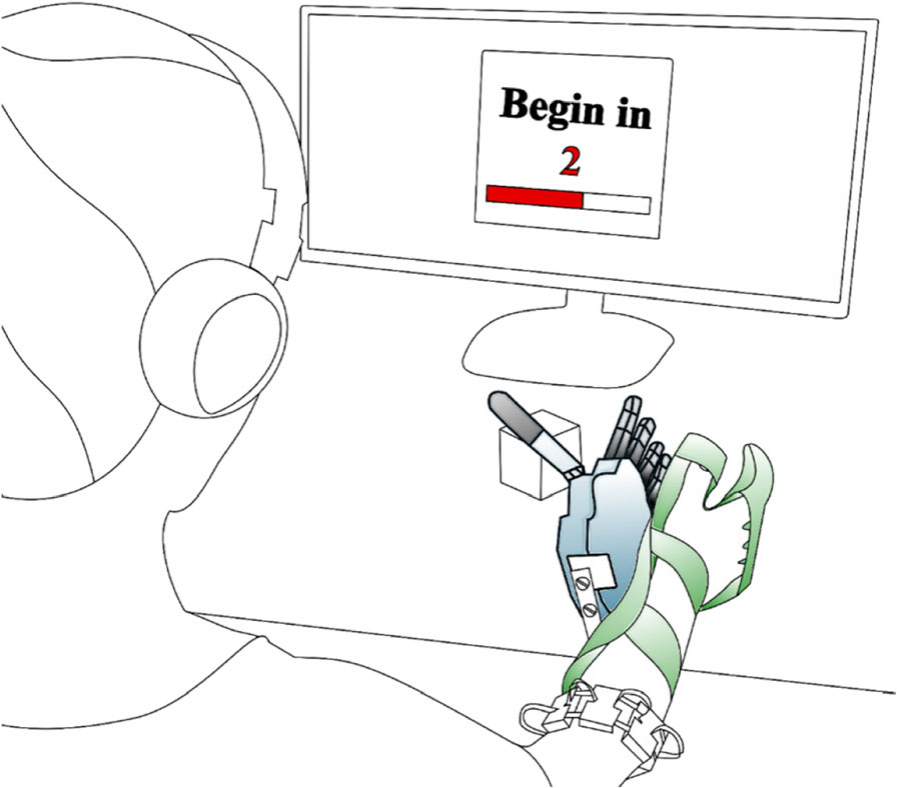
Subject controlling a prosthetic hand to grasp-and-lift an instrumented virtual egg without breaking it. The prosthetic hand is controlled using the subject’s myoelectric signals sensed by an electrode array placed on their forearm

The experimental platform consisted of a robotic hand, an array of myoelectric sensors, a PC implementing the control strategy, headphones that conveyed audio feedback, and a test object instrumented with force sensors (Fig. 2). The robotic hand was a right-handed version of the IH2 Azzurra Hand (Prensilia, IT) [44]. It consists of four fingers and a thumb actuated by five motors. In the present work, movements were limited to allow only flexion/extension of the thumb-index-middle digits and the abduction/adduction of the thumb. The hand included encoders on the motors, which were under position control based on commands sent over a serial bus from the PC. Subjects controlled the robotic hand using isometric muscle contractions sensed by an array of eight low power multi-channel operation electrodes (30 × 20 × 10 mm/electrode) placed around their forearm [48]. Seven subjects tested the classifier-based control without augmented feedback (NF) and then retested with the audio-augmented feedback (AF). The remaining subjects tested the classifier-based control without augmented feedback (NF) twice to test for learning effects.

The test object was an *instrumented Virtual Egg* (iVE). The iVE is a rigid plastic test-object (57 × 57 × 57 mm^3^; approximately 180 g) equipped with two strain gauge-based force sensors (Strain Measurement Devices, UK, model S215–53.3 N; each located at one of two parallel grasping sides), able to measure grip force exerted on the object. The iVE was programmed to virtually break whenever the grip force was larger than a preset threshold (approximately 3.1 N); this event was signaled to the subject through a colored light on the iVE [45].

### Protocol

Participants were instructed to repeatedly grip, lift, replace, and release the iVE at a self-selected routine grasping speed. Specifically, their task consisted of (1) moving their right arm to reach the iVE with the robotic hand mounted on a bypass splint (Fig. 1), (2) contracting their own muscles to control the robotic hand so that it grasped the object, (3) lifting the iVE a few centimeters above the table, (4) putting the iVE back on the table, and, finally, (5) releasing the object by opening the hand.

During the experiment, subjects sat comfortably in front of a computer screen and wore a set of 1000 mW headphones (MDRZX100, Sony, JP) with the volume set to a maximum of 52.5 ± 3 dB (they could remove them during scheduled breaks between testing blocks). Subjects used each feedback method to complete a series of test blocks in a specific order after accomplishing a training and familiarization block. Before the start of each test block, subjects were given a two-minute break, in which they could stand up, remove the headset, unstrap the splint, and stretch if needed. The electrode array, however, was not removed for the duration of the experiment.

**Fig. 3:**
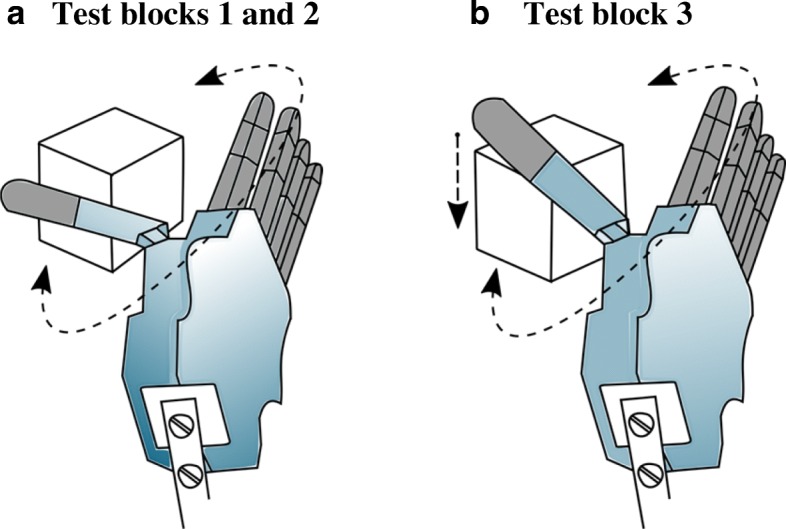
Hand starting pose. **a** Starting pose for the training and familiarization, adaptation, and JND blocks. Subjects had to only activate the thumb and fingers flexion to grasp the object carefully without breaking it. **b** Starting pose for the performance test: fingers and thumb are extended, and the thumb is abducted. Subjects had to adduct the thumb and then close the hand to grasp the object and transfer it from one side of a barrier to the other

The training and familiarization block consisted of 40 grasp-and-lift trials. Subjects were given verbal instructions to complete the task without breaking the iVE in less than seven seconds after which a “Time out” text appeared on the computer screen and the artificial hand returned to a predetermined starting pose (Fig. 3). The training and familiarization starting pose was with the hand fully opened and thumb adducted (Fig. 3a). While in the first 25 trials, subjects were shown the feedback when the iVE broke (fragile mode), they were not given this feedback in the last 15 trials (rigid mode). This was done to keep subjects engaged with the task and not lose interest during the training block. Subjects were allowed to proceed to test blocks when they achieved at least 75% successful grasp-and-lift trials of the iVE in the training block.

The first test block was used to test adaptation to self-generated errors. In this block, subjects were asked to complete 40 grasp-and-lift trials in less than five seconds per trial. Adaptation rate was computed as how much subjects adjusted their grasp trajectory from one trial to the next based on error observed between their actual trajectory and activating only the hand close/open DOF, i.e., the optimal trajectory [49].

The second test block was used to measure the subject’s perception threshold, i.e. a psychometric measure of sensory threshold for perception of a sensory stimulus. Subjects performed a series of two lift trials (fragile mode). In one of the two trials, a specific stimulus was added causing the hand to behave differently. The subjects were then asked to identify the changed trial by pressing the “1” or “2” key (for trial one or two) on a keyboard placed in front of them with their other hand. The magnitude of the added stimulus was calculated using an adaptive staircase as a rotation in the control space in degrees (Fig. 3 in [38]) with target probability set to 0.84 [50, 51]. For instance, if a subject was generating control signals for thumb abduction, a 90 degrees rotation in the control signal would switch activations from thumb abduction to hand close. Each trial lasted for four seconds and subjects were encouraged to take breaks between trials whenever they needed. The final noticeable stimulus reached was recorded when the number of reversals for this staircase reached 23 [38]. The starting pose of the prosthetic hand for the first and second block was similar to that of the training and familiarization block where subjects had to only activate the hand close/open DOF to achieve this task efficiently.

The third and last test block was used to measure performance. Subjects were given 20 trials to move the iVE (fragile mode) from one side of a barrier (H: 14.5 cm x W: 25 cm) to the other in less than 10 s per trial, similar to the Box and Blocks test [52]. The starting pose of the hand was adjusted to evaluate the subject’s performance for a 2-DOF task in which subjects had to activate the thumb adduction/abduction DOF to lower the thumb and then activate the hand close/open DOF to grasp the iVE properly to lift it to the other side of the barrier (Fig. 3b). Table 1 summarizes the experimental protocol used in this study.

**Table 1:** Summary of the experimental protocol

Task	Description
Control Practice	Control the prosthetic hand for two minutes – a combination of close/open the prosthetic hand and abduct/adduct the thumb.
Training and Familiarization	25 trials of grasp-and-lift of the iVE with the breaking feedback and 15 grasp-and-lift trials without the breaking feedback. Each trial lasted for seven seconds.
Adaptation rate test	A total of 40 grasp-and-lift trials. Each trial lasted for five seconds.
Perception threshold test	Grasp-and-lift the iVE in less than four seconds and identify the trial with the added stimulus in a set of two trials, repeat this task until convergence of an adaptive staircase.
Performance test	Transfer the iVE from one side of a barrier to the other 20 times in less than 10 s per transfer.

### Outcome measures

#### Internal model parameters

Similar to our previous research [38, 43], we assessed human understanding of the myoelectric control strategy for a grasp-and-lift task using the following psychometric measures:

- Adaptation rate (−*β*_1_) is a measure of feedforward modification of the control signal from one trial to the next [37]. For each trial in the adaptation rate test, control signal activations in both DOFs, i.e., flex/extend thumb-index-middle digits and adduct/abduct thumb, were recorded. To capture the subject’s feedforward intent, the first 500 ms of the recorded activations for each trial were analyzed. The target control signal was the activation of the closing of the prosthetic hand only (i.e., flex/extend thumb-index-middle digits). Other activations were considered as self-generated errors, which subjects were instructed to minimize. The following equation was used to compute this adaptation rate.

1 $$ {error}_{n+1}-\kern0.5em {error}_n\kern0.5em =\kern0.5em {\beta}_1\kern0.5em \times \kern0.5em {error}_n\kern0.5em +\kern0.5em {\beta}_0 $$

where *error* is the angle formed between the closing of the hand activation trajectory, i.e., target, and the actual hand activation trajectory; *n* is the trial number; *β*_0_ is the linear regression constant; and −*β*_1_ is the adaptation rate. A unity value indicates perfect adaptation, i.e., internal model modified to perfectly compensate for errors.

- Just-noticeable-difference (JND) is a measure of the minimum perceivable stimulus in degrees identified by the subject when using each feedback method [50]. A lower threshold indicated better user ability to perceive small perturbations in the control strategy used. This parameter was extracted from the Perception threshold test block as the final noticeable stimulus when the number of reversals for an adaptive staircase reached 23.
- Internal model uncertainty (*P*_*param*_) is a measure of the confidence of a user in the internal model they developed for a control strategy with a certain feedback method. This parameter was computed using outcomes from both the first and second test blocks [38].

#### Performance parameters

- Completion Rate (CR) is the percentage of the successful transfers of the iVE from one side of the barrier to the other without breaking it (fragile mode). This parameter was extracted from the third test block.
- Mean Completion Time (MCT) is defined as the time taken to successfully transfer the iVE from one side of the barrier to the other without breaking it (fragile mode). This parameter was also extracted from the third test block.
- Trial submovements (TS) is the number of submovements per trial. This parameter is calculated as the number of zero-crossing pairs of the third derivative of the grasp force profile per trial [53]. The number of submovements served as an indicator of use of feedback for real-time regulation of the grasping force [54–56]. The higher this number, the greater the use of feedback in real-time regulation. This parameter was extracted from the adaptation test.

### Statistical analysis

The Statistical Package for the Social Science software (SPSS v25.0, IBM, US) was used to run Levene’s test on JND, adaptation rate, internal model uncertainty, and performance measure results to investigate homogeneity in variances of the data. If data variances were found to be homogenous, we ran two-sample paired t-tests to assess differences between these outcome measures at a significance criterion of α = 0.05 for the two feedbacks tested. If data variances were found to be nonhomogeneous, a Wilcoxon signed-rank test was conducted. For the group of subjects who tested and retested the NF controller, repeated measures ANOVA was used to compute intraclass correlation coefficient (ICC) for internal model parameters and performance parameters using a two-way mixed effects model with absolute agreement at a 95% confidence interval to investigate the effect of prolonged exposure to a control strategy. The confidence interval was calculated using the standard deviation (95% CI = mean ± 1.97 × SD). If not denoted otherwise, all numbers in the text refer to mean ± SD.

## Results

To confirm that the benefits of using audio-augmented feedback for improving internal model strength of myoelectric controllers extend beyond a virtual target acquisition task [43], we assessed the internal model developed when using this audio-augmented controller and the no-augmented feedback controller to control a prosthetic hand for a grasp-and-lift task. In addition, short-term performance when using both controllers was evaluated.

### Internal model assessment

Two psychophysical experiments were employed to evaluate parameters that are used to assess internal model strength [38]. The first experiment tested the trial-by-trial adaptation to self-generated errors. The outcome from that test indicated how much the internal model was modified from one trial to the next based on error feedback.

**Fig. 4:**
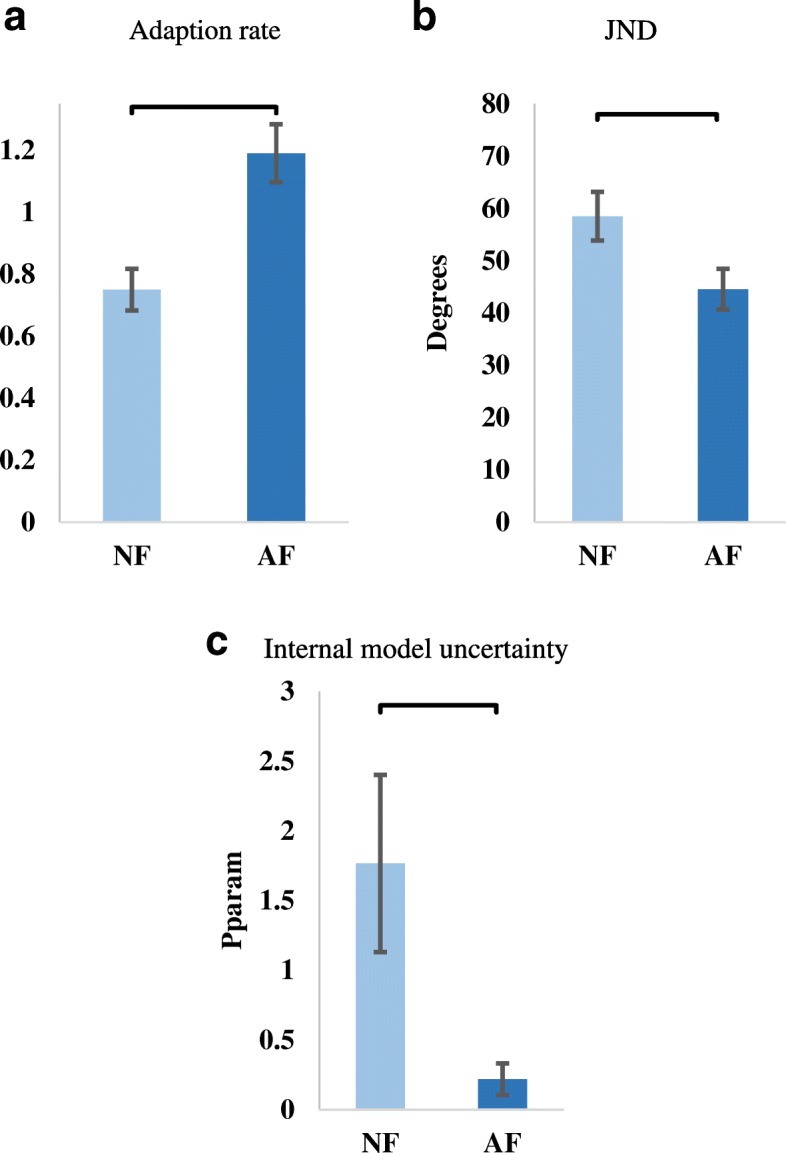
Psychophysical test results. **a** Adaptation rate results showing audio-augmented feedback control strategy enabling higher adaptation to self-generated error than the no-augmented feedback control strategy. **b** Perception threshold test results showing low JND value when using the audio-augmented controller. **c** Internal model uncertainty (Pparam) results showing significant reduction in the internal model uncertainty when using the audio-augmented feedback control strategy. Horizontal bars indicate statistical significant difference. NF: No-augmented Feedback. AF: Audio-augmented Feedback

Results from the adaptation test (first test block) proved a statistically significant difference between subjects using NF and AF control strategies (two paired-samples t-test (t (6) = − 4.6), *p* = 0.004)). In particular, the AF control strategy promoted a significantly higher adaptation rate (1.2 ± 0.25) than the NF control strategy (0.75 ± 0.15) (Fig. 4a).

The outcomes from the perception threshold test matched with those from the adaptation test. Audio-augmented feedback control strategy enabled a significantly lower perception threshold (44.6 ± 10 degrees) than the NF controller (58.5 ± 12.5 degrees) (paired samples t-test (t (6) = 3.4, *p* = 0.014)) (Fig. 4b).

The adaptation rate and the JND were used to compute the internal model uncertainty developed for each of the tested feedback conditions. Again, the AF control strategy promoted a lower internal model uncertainty (0.22 ± 0.11) compared to subjects using NF control strategy (1.8 ± 0.6) (related samples Wilcoxon signed-rank test; *p* = 0.018) (Fig. 4c).

Test-retest of NF controller: Results for internal model assessment parameters showed no significant within-subject effect of retesting NF controller with good reliability (ICC > 0.65). Table 2 summarizes the statistical analysis for these results.

**Table 2:** Summary of test-retest for the Nf controller results

Outcome measure	ANOVA repeated measure p	ICC	SEM
Adaptationrate	0.86	0.65	0.102
Just-noticeable-difference	0.21	0.65	4.4
Internal model uncertainty	0.64	0.9	0.53
Completion Rate	0.57	0.9	1.2
Mean completion time	0.47	0.55	0.16

All in all, these results align with previous studies [43] and confirm that audio-augmented feedback promotes: (1) high adaptation rate, (2) the user’s ability to perceive low sensory threshold and, in turn (3) a strong internal model for a grasp-and-lift task using a prosthetic hand.

### Performance test

**Fig. 5:**
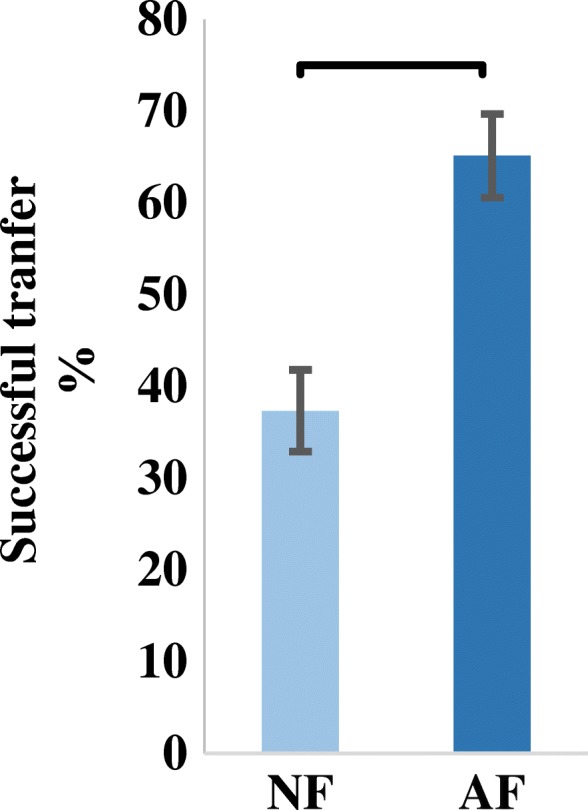
Successful transfer rate of the instrumented virtual egg from one side of a barrier to the other without breaking it. Subjects had 1.74 times higher successful transfers when using the audio-augmented feedback control strategy than when using the no-augmented feedback control strategy. NF: No-augmented Feedback. AF: Audio-augmented Feedback

**Fig. 6:**
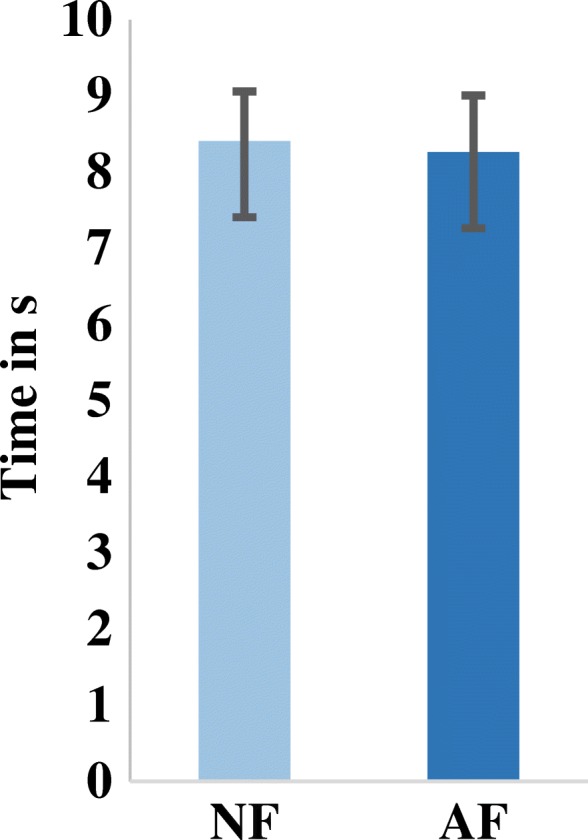
Completion time for successful transfers. Subjects using the no-augmented feedback controller had similar completion time to subjects using the audio-augmented controller. NF: No-augmented Feedback. AF: Audio-augmented Feedback

The completion rate (in the last test block) proved higher when using the AF control strategy (65 ± 12%) than when using the NF control strategy (37.34 ± 19%) (Two paired-sample t-test, (t (6) = − 2.87, *p* = 0.028) (Fig. 5). Notably, testing of the mean completion time did not exhibit a significant difference (MCT_AF_ = 8.3 ± 0.74 s; MCT_NF_ = 8.4 ± 0.65 s) (Fig. 6).

Test-retest of NF controller: Similar to the internal model assessment parameters results, results for performance parameters showed no significant within-subject effect of retesting the NF controller with very good reliability (ICC > 0.9, CR) and good reliability (ICC = 0.55, MCT) (Table 2).

**Fig. 7:**
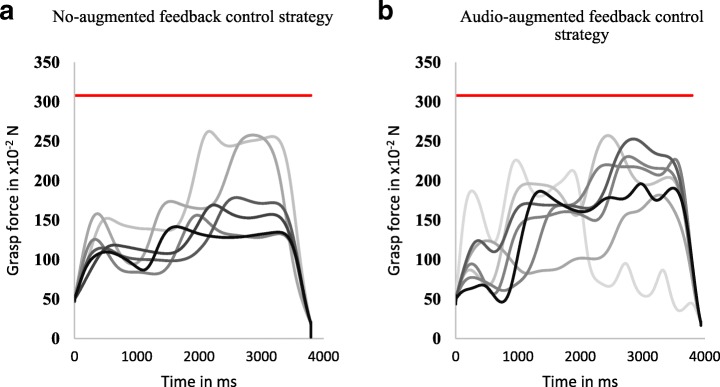
Progression of grasp-and-lift trials ranging from the beginning of the task (light gray) to the end of the task (dark gray). Representative data from a single subject during adaptation rate test using (**a**) the no-augmented feedback control strategy (moderate grasp force changes per trial) and (**b**) the audio-augmented feedback control strategy (high grasp force changes per trial). The red line in both plots shows the preset breaking force

**Fig. 8:**
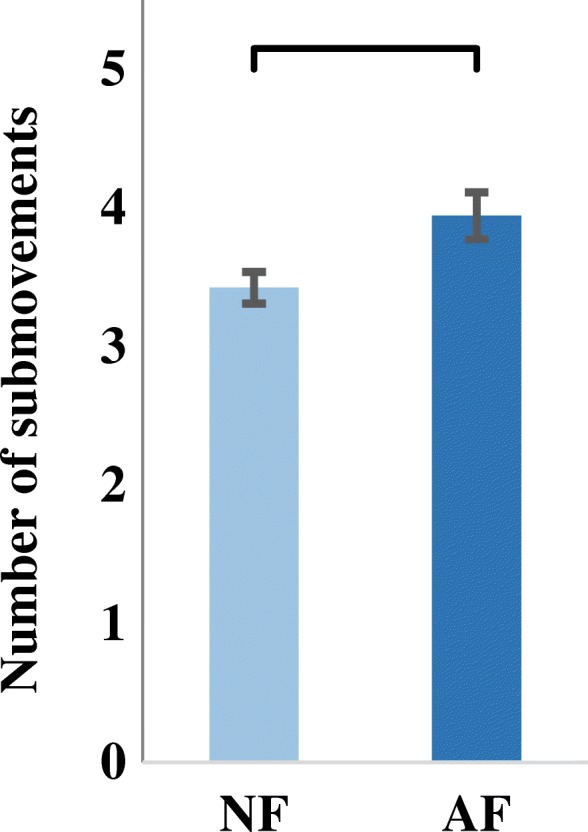
Submovements computed from the grasp forces of successful trials from the adaptation rate test for a sample of five subjects. NF: No-augmented Feedback. AF: Audio-augmented Feedback

Submovements analysis was performed on data recorded from only five subjects as the iVE failed to record data for the other two subjects due to a communication error. When using the NF control strategy, subjects changed their grasping force during the grasp-and-lift task, though not as much as when using AF control strategy (Fig. 7). Results show that subjects using AF control strategy had a significantly higher number of submovements (3.94 ± 0.12) than subjects using NF control strategy (3.26 ± 0.17) as determined by a two-sample independent t-test (t (90) = − 3.17, *p* = 0.002) (Fig. 8). These results suggest that audio-augmented feedback enables better short-term performance by enabling the development of a stronger internal model.

## Discussion

Many studies have focused on improving performance of myoelectric prosthesis control by providing feedback to the user, but only a few have investigated the effect of this feedback on the internal model, which is key to improving long-term performance [57]. Due to an inability to assess internal model strength, this effect remained unquantified. For the first time, we used a recently developed psychophysical framework to assess the strength of the internal model developed when using different myoelectric prosthesis controllers [38]. In earlier work, we demonstrated that audio-augmented feedback improves internal model strength and the performance of myoelectric prosthesis control in a virtual target acquisition task [43]. We argued that these improvements may extend beyond a virtual target acquisition task. In this study, we tested the classifier-based control with and without audio-augmented feedback for a grasp-and-lift task when using a prosthetic hand. Our results confirm the hypothesis that audio-augmented feedback enables the development of a strong internal model and better short-term performance when controlling a prosthetic hand for a grasp-and-lift task.

Even when using different controllers, humans are able to incorporate previous knowledge and experience to accomplish tasks [38]. To minimize this translation of stronger internal models, all subjects in this study tested the no-augmented feedback controller first, followed by the augmented audio feedback controller. It could be possible that the reduction in internal model uncertainty for the audio-augmented controller is due, in part, to the prolonged exposure to the control strategy and the experiment. This possibility, however, was addressed in this work when subjects were asked to test and retest the same control strategy (no-augmented feedback) and it was concluded that there was no improvement in adaptation rate, JND, internal model strength, or performance due to the repetition of the test. Consequently, we argue that any improvement in those parameters is due to the control strategy used and not due to prolonged exposure.

To ensure that the continuous audio feedback was not a distraction to the user and, in turn, did not compromise short-term performance, we assessed the short-term performance by computing the completion rate (without breaking the object). Outcomes, in fact, showed a significantly better performance when subjects used the AF compared to the NF controller, albeit both controllers had similar completion time. The submovements analysis revealed that subjects adjusted their grasping forces more frequently when using the AF controller than when they used the NF controller. This finding suggests that augmented audio feedback may not only be used for developing internal models, but subjects’ high confidence in the feedback lead to them using this feedback for real-time regulation too. Hence, regression-based augmented audio feedback improves both short-term performance through real-time regulation and long-term performance through development of strong internal models.

Although we did not measure the cognitive load of using audio feedback in this work, other researchers have found that audio feedback may be used to alleviate the cognitive burden when combined with visual feedback [25]. Internal model assessment results from this study may be used to further explain how audio feedback reduces the cognitive load. To further support our findings, future work may include utilizing visual attention measures developed in [6] to quantitatively determine the effect of using controllers with and without feedback on visual attention.

Although the results found in this study providing compelling evidence that internal models can indeed be improved using augmented feedback, they must still be confirmed in the target population. Although we tested only able-bodied subjects, we suspect that similar internal model results may be found when testing amputees since internal model assessment parameters are measures of human behavior and understanding and not physical ability [39]. That said, performance results found here may be scaled when testing amputees due to differences in prosthesis attachment, i.e., bypass vs. socket, and placement of the surface electrode array. One might argue that the control strategy (model) trained and used by able-bodied subjects in this study will be very different than the one trained and used by an amputee. In fact, the control model trained for every individual and for every session is unique and tuned to that individual regardless of chosen location of the electrode array or muscle mass. This trained model is driven by how individuals contract their muscles for a given DOF model training. The length of the residual limb available for electrode placement and integration of sensory feedback in the socket are indeed challenges that are not faced when testing able-bodied subjects and must be addressed when testing amputees. It must be noted that the use of the audio feedback modality in this work reduces the challenges associated with integrating sensory feedback mechanisms within the socket.

In this study, we conducted psychophysical tests on one DOF, i.e., closing the hand to grasp-and-lift an object, to avoid fatigue and loss of motivation. However, we designed the performance test for a two-DOF task where subjects had to activate both DOFs, i.e., digits flexion/extension and thumb adduction/abduction, to ensure that they were able to fully control the device to achieve the task and to collect performance results that could be compared to previous studies [38, 42, 43]. During the performance test, we noticed that lifting the weight of the prosthetic hand affected users’ ability to open the hand after grasping the object, which affected the performance for both control strategies tested equally. This weight effect could be avoided in future experiments by using a tool balancer [58].

Furthermore, some subjects reported that continuous audio feedback may be a distraction; however, our results show that, although subjects may not purposely focus on integrating this feedback, they unconsciously integrate it into their internal models. With this in mind, a new question arises: would a task specific discrete audio feedback, i.e., discrete beeps on contact and release of an object akin to the Discrete Event-driven Sensory feedback Control (DESC) principle [1, 59], be less irritating while potentially enabling similar integration? This question will be addressed in future research.

Although audio-augmented feedback showed promising results, the minimum quantity of feedback that is useful for developing strong internal models must still be identified, along with what quality is required for real-time regulation. Future work informed by this study includes: investigating the benefits of using audio feedback for limb-different individuals, exploring a combination of other augmented feedback that might enable an even stronger internal model, exploring the effect of augmenting other feedback modalities on the internal model strength, investigating the effect of audio-augmented feedback control strategy for a more complex task on the internal model strength and the performance, and, finally, investigating the retention of internal models developed while using the audio-augmented feedback control strategy.

## Conclusions

We extended our previous work to investigate the benefits of using audio-augmented feedback by testing a classifier-based control with and without this feedback for a grasp-and-lift task when using a prosthetic hand. Results from psychophysical and performance tests showed that audio-augmented feedback enables the development of a strong internal model and better short-term performance. In addition, we concluded that audio feedback may be used in real-time regulation of grasping forces during a grasp-and-lift task.

## Abbreviations

AF: Audio-augmented Feedback
CNS: Central Nervous System
CR: Completion Rate
DESC: Discrete Event-driven Sensory feedback Control
DOF: Degree of Freedom
EMG: Electromyography
iVE: instrumented Virtual Egg
JND: Just-Noticeable-Difference
MCT: Mean Completion Time
NF: No-augmented Feedback
SVR: Support Vector Regression
TS: Trial Submovements
